# Patient assignment optimization in cloud healthcare systems: a distributed genetic algorithm

**DOI:** 10.1007/s13755-023-00230-1

**Published:** 2023-06-29

**Authors:** Xinyu Pang, Yong-Feng Ge, Kate Wang, Agma J. M. Traina, Hua Wang

**Affiliations:** 1 Guangdong Technion Israel Institute of Technology, Shantou, China; 2 Institute for Sustainable Industries and Liveable Cities, Victoria University, Melbourne, Australia; 3 School of Health and Biomedical Sciences, RMIT University, Melbourne, Australia; 4 Institute of Mathematics and Computer Sciences, University of São Paulo, São Paulo, Brazil

**Keywords:** Patient assignment, Distributed genetic algorithm, Cloud healthcare system, Evolutionary algorithm

## Abstract

Integrating Internet technologies with traditional healthcare systems has enabled the emergence of cloud healthcare systems. These systems aim to optimize the balance between online diagnosis and offline treatment to effectively reduce patients’ waiting times and improve the utilization of idle medical resources. In this paper, a distributed genetic algorithm (DGA) is proposed as a means to optimize the balance of patient assignment (PA) in cloud healthcare systems. The proposed DGA utilizes individuals as solutions for the PA optimization problem and generates better solutions through the execution of crossover, mutation, and selection operators. Besides, the distributed framework in the DGA is proposed to improve its population diversity and scalability. Experimental results demonstrate the effectiveness of the proposed DGA in optimizing the PA problem within the cloud healthcare systems.

## Introduction

The rapid advancement of Internet and information technologies [1–7] has led to a growing demand for cloud healthcare systems [8–13] that can effectively provide all medical services [14–16]. These systems are based on integrating online diagnosis [17–21] and offline treatment to reduce patients’ waiting time and improve the utilization of idle medical resources. However, the development of such systems [22–25] is contingent upon the successful resolution of the patient assignment (PA) problem. The PA problem is a crucial aspect of cloud healthcare systems, as it directly impacts the efficiency and effectiveness of the system. Therefore, it is paramount that the PA problem is carefully considered and appropriately addressed in the design and implementation of cloud healthcare systems.

The PA problem in cloud healthcare systems has been the subject of ongoing research, with various strategies proposed to address it. One such approach is the use of discrete event simulation to develop a queuing model [26]. This strategy aims to reduce patient waiting time and increase the system’s overall throughput. Another approach uses Petri nets to describe the relationship between medical processes and resources [9]. A hybrid ant agent algorithm has also been proposed [27], which aims to identify the optimal path for patients, thus reducing both waiting and cycle time. Previous studies have emphasized the importance of reducing patients’ waiting time. However, it should be noted that a continuous influx of patients characterizes cloud healthcare systems. The balance of assignments among doctors is also crucial in improving the system’s efficiency. Therefore, in this paper, we optimize the balance of assignments among doctors in the cloud healthcare systems.

The optimization of the PA problem can be achieved through the utilization of genetic algorithms (GAs) [28, 29]. GAs are a type of evolutionary algorithm (EA) [30–33] that have been widely used in the field of computational mathematics to solve optimization problems. Evolutionary biology concepts such as heredity, mutation, natural selection, and hybridization are used to construct EAs [34–36]. GAs are beneficial for finding reasonable solutions quickly, even in complex spatial solutions, by using parallel studies, selection operations, alteration operations, and mutation functions [37–39]. Previous studies have demonstrated the advantages of using EAs, including GAs, in various scenarios, such as reliability and performance. They have been applied to various fields such as computer science, engineering, and operations research and have consistently shown to be effective in solving optimization problems [40–42]. Previously, GA has been utilized in the optimization of the PA problem [43] and its advantages in terms of convergence speed and scalability have been verified.

This paper proposes a distributed genetic algorithm (DGA) to optimize the PA problem. Over the previous approaches for the PA problem, DGA shows its advantages of global optimization performance and diversity maintenance (not easily trapped by local optima), robustness and scalability (the capability of handling complex and noisy problem spaces), flexibility (easily fits different problem formulation), and increased parallelism (enables faster convergence and reduces running time). Each individual in the proposed DGA represents a solution to the PA optimization problem. Several individuals in the proposed DGA form multiple sub-populations. During the evolution of each sub-population, information included in all the individuals is exchanged by the crossover operator. Individuals are randomly adjusted in the mutation operator. After that, the selection operator evaluates the competitiveness of different solutions. The more competitive solutions are kept in the population, and the less competitive individuals are gradually eliminated. Then, with a predefined interval, the elite individuals of all the sub-populations are exchanged to accelerate the convergence. Finally, the optimal solution to the PA problem is outputted.

More specifically, the contributions of this paper are listed as follows.

1. We propose the DGA to optimize the PA balance in cloud healthcare systems.
2. We propose a distributed framework in the DGA to improve population diversity and scalability.
3. We utilize the operators in DGA to improve the competitiveness of the solutions to the PA problem.

The organization of this paper is as follows. In Sect. 2, a formal problem formulation of the PA problem is illustrated. Then, we review the related work of the PA problem and the application of GA. In Sect. 4, we introduce the DGA. Afterward, the proposed DGA is introduced in detail. In Sects. 6 and 7, the experimental study is executed, and the experimental results are analyzed. Finally, we conclude this paper.

**Fig. 1:**
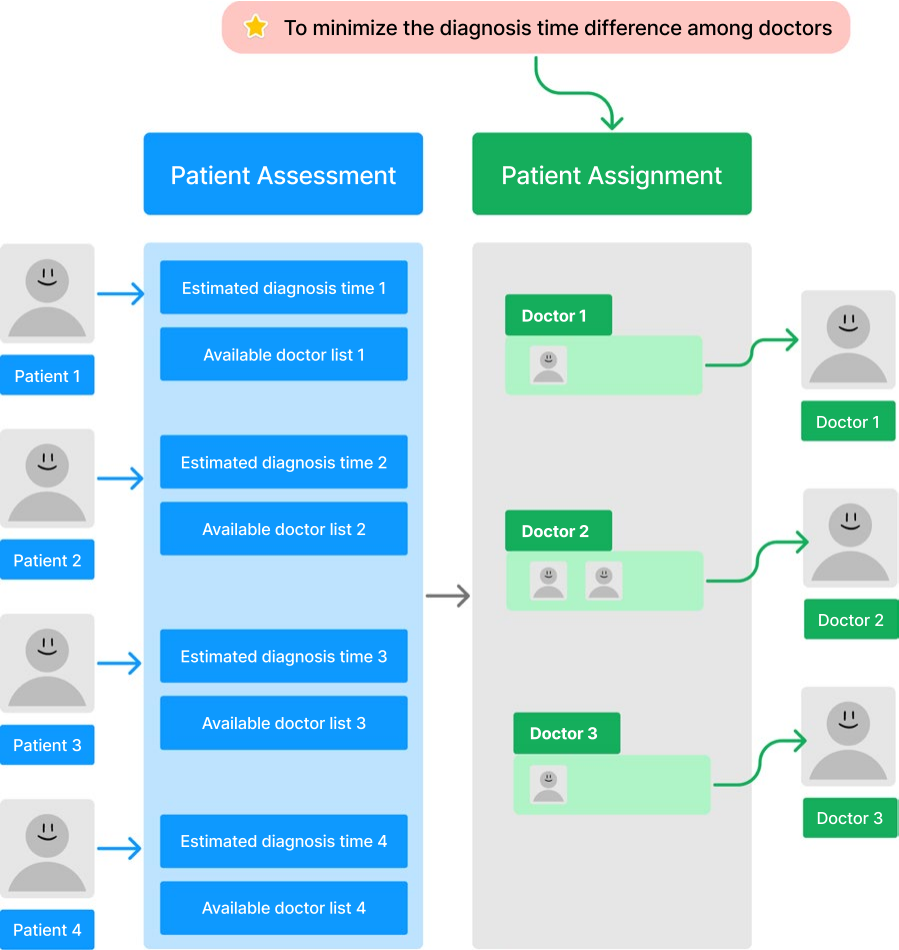
Illustration of the patient assignment in the cloud healthcare systems

## Problem formulation

In Fig. 1, an example of patient assessment and assignment modules is given. In the given example, the condition of four patients is assessed at the beginning. Accordingly, the estimated diagnosis time and available doctor lists are produced. Our optimization objective in the patient assignment module is to minimize the diagnosis time difference among different doctors. Finally, the patients are assigned to the corresponding doctors for further diagnosis.

Specifically, in PA, the *i*-th patient is represented by $P_i$; $D_j$ represents the *j*-th doctor. The estimated diagnosis time of *i*-th patient is indicated by $\widetilde{T_i}$.

The total diagnosis time of *j*-th doctor (represented by $T_j$) is calculated as follows:

1 $$\begin{aligned} T_j=\sum _{i=1}^{nP} \widetilde{T_i}\times S_i^j \end{aligned}$$

where *nP* is the number of patients; S indicates a status matrix. $S_i^j$ equals to one when the *i*-th patient is allocated to the *j*-th doctor; $S_i^j$ equals to zero when the *i*-th patient is not allocated to the *j*-th doctor.

Thus, the mean value of diagnosis time is calculated as:

2 $$\begin{aligned} \overline{T}=\sum _{j=1}^{nD} T_j \end{aligned}$$

where *nD* is the number of doctors.

The time factor (TF) is then obtained by calculating the standard deviation of diagnosis time of all the doctors. Formally,

3 $$\begin{aligned} \text {TF}=\sqrt{\frac{\sum _{j=1}^{nD}(T_j - \overline{T})^2}{nD}} \end{aligned}$$

As mentioned above, the optimization objective is to balance the doctors’ diagnosis time. Therefore, the optimization objective is to minimize the value of TF.

## Related work

In [44], a positive model of the public hospital waiting lists was established. According to the studies, doctors did not necessarily treat the mildest cases on the waiting list to have the shortest overall hospital stay. In [45], Takakuwa and Wijewickrama created a discrete-time simulation model and integrated the simulation model into the optimization algorithm to reduce patient waiting and physician idle time without adding any additional resources. In [46], the dynamic patient scheduling with different priorities in a public healthcare setting was tackled. The proposed method dynamically assigns available capacity to incoming demand to achieve cost-effective wait-time targets. This study collected real-time data from Nagoya University Hospital’s outpatient clinic to create a simulation. In [47], the Lean Six Sigma (LSS) method was used to solve the problem of the long waiting time of patients. The entire procedure was covered, from patient registration to prescription distribution. A causal map was created for patients with longer waiting times, and data collected during the process were used to verify the reasons. In [26], a queuing model was developed using discrete event simulation, which could reduce the patient waiting time and improve the system’s overall throughput. To resolve ambiguities in the present system, required data was collected, and alternative scenarios were generated and examined. Furthermore, the best solution concerning patient satisfaction was proposed. In [48], a system was designed to reduce the doctors’ idle time instead of the patients’ waiting time. It provides an alternative perspective on this problem. This study aimed to improve resource efficiency and modify how doctors schedule visits. The results showed that patients’ waiting time might be lowered without affecting doctors’ work efficiency. In [9], a Petri net was presented to describe the relationship between the medical process and resources in this integrated healthcare system. A PA scheduling problem was investigated and studied to allocate this system’s bottleneck medical resource efficiently. A mathematical model was established, and a greedy-based heuristic algorithm was designed. In [49], Chawasemerwa et al. developed a constraint satisfaction and penalty minimization scheduling model that satisfied “hard constraints” and minimized the cost of “soft constraints” violations. Furthermore, since multiple schedules may be obtained using the same parameters defined by users, an optimization protocol can be added to the system to reduce the search space and obtain the optimal schedule while satisfying the constraints. In [27], the real-time walk-in patient scheduling optimization problem was addressed. An overall patient scheduling model was integrated. The status and information of all outpatient departments were combined. The hybrid and agent algorithm was developed to identify the best path for the patient while also lowering cycle time (from registration to exit). In [50], similar issues have been further refined. Conforti et al. defined that the scheduling objective of radiotherapy patients in the oncology department was to ensure the best treatment in the shortest possible time. As a result, the waiting time should be minimized, and device utilization should be maximized. Various criteria were added to the optimization model.

The limitations of previous PA approaches are manifold. Firstly, previous PA approaches emphasized the importance of reducing patients’ waiting time, ignoring the balance of assignments among doctors, which is crucial for cloud healthcare systems’ efficiency and scalability. Secondly, previous optimization approaches did not provide sufficient global optimization performance, easily trapped by the local optima. Thirdly, no distributed computation framework was proposed. Therefore, the convergence speed is limited and the running time cannot be reduced.

The application of GAs also has remarkable achievements in the medical and healthcare fields. Yadav et al. [51] focused on optimizing blood bank inventory control, a healthcare system, on enhancing its determinism. The problems of inbound and outbound logistics and inventory inflation were solved by a multi-objective GA and reliability application using minimum cost optimization of other parameters. Ahmed et al. [52] improved the modeling of building degradation to alleviate budgetary constraints on the maintenance of medical resources and to reduce the incidence of accidents. Developing a fuzzy Markov model based on a hybrid GA with a nonhomogeneous transition probability matrix based on fuzzy membership functions representing the hospital system’s condition, age, and relative deterioration rate is utilized to address the inherited uncertainties. Mutingi and Mbohwa [53] tackled the home healthcare worker scheduling problem. Considering the accelerating demand for home care requires careful task allocation and scheduling of limited healthcare resources, Mutingi and Mbohwa proposed a group GA for scheduling the dispatch of healthcare while considering the minimum economic cost of time.

## Genetic algorithm

GAs are potent meta-heuristics that improve and refine Darwin’s theory of natural evolution. Based on initialization methods, GAs usually start by constructing initial populations in a randomized and uniform manner. Each population is evaluated for its fit to the target problem using a fitness function. New populations are formed through a series of processes, such as crossover and mutation, and new individuals replace the original ones to form a new population. The great advantage of GAs is that they can process problems of different dimensions in parallel, considering several factors and characteristics simultaneously. It is possible to optimize the computational speed by managing the task allocation between off-the-shelf. In terms of application areas and problem areas, we focus on the typical characteristics of applications and the classification of GAs, respectively, and optimize solutions from different dimensions through examples. The overall procedure of GA is given as follows.

First, the program creates a set number of individuals representing the solutions to the optimized problem at random. When the operator interferes with this randomly produced process to increase the quality of the first population, the quality of the initial population improves. After that, each generation’s individuals are given a value, and the fitness value is calculated using the fitness function. Dominant populations obtain a higher degree of adaptation compared to disadvantaged populations.

The next step is to generate the next generation of individuals to form the population. This process is done by selection and replication, which involves crossover and mutation in algorithmic studies. Selecting the winners from the population and eliminating the inferior ones is called selection. The goal of selection is to pass on their directly optimized genes to the next generation or generate new individuals through crossover pairing and generation, which are then passed on to the next generation. Selectivity is based on assessing the individual’s physical condition in the population. Selection is based on the fitness of new individuals. However, it does not mean at the same time that it is entirely oriented toward fitness because simply selecting individuals with high fitness will lead to a rapid local conversion of the algorithm to the optimal solution rather than to the optimal global solution, which we call the initial stage. As a compromise, GAs follow the principle that the higher the fitness, the higher the chance of being selected, and the lower the fitness, the lower the chance of being selected. The initial data can be selected to form a relatively optimal group.

After that, the selected individuals enter the mating process. The core of biological evolution in nature is the recombination of biogenetics (coupled with mutation). After this series of processes (selection, crossover, mutation), a new generation of individuals differs from the first generation. Each generation moves toward improved overall fitness Because individuals with greater adaptability are more likely to survive and produce the next generation. Conversely, poorly adapted individuals are gradually eliminated.

## Distributed genetic algorithm for patient assignment

This section illustrates the proposed DGA for optimizing the PA problem. Firstly, we introduce the representation manner and initialization strategy of DGA. Secondly, the distributed framework of DGA is illustrated in detail. Afterward, the crossover and mutation operators of DGA are described. Finally, the entire procedure of DGA is described.

**Fig. 2:**
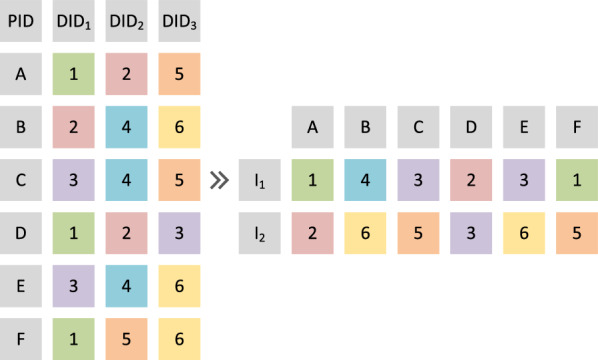
Illustration of the representation manner in DGA

### Representation and initialization

In GA, each individual represents a solution for PA. In each individual, each gene indicates the assignment of each patient. An example of this representation manner is given in Fig. 2. In this example, three doctors allocated to for each patient (represented by A, B,..., F). Different digits with different colors represent different doctors. In total, six doctors are included in this example. Therefore, one doctor is chosen from the candidature lists for each patient. In this example, two individuals are given (represented by $\mathrm {I_1}$ and $\mathrm {I_2}$). For the first patient (patient A), doctor 1 is allocated to in individual $\mathrm {I_1}$, while doctor 2 is allocated to individual $\mathrm {I_2}$. For each complete individual, it can be directly evaluated according to the definition of the PA problem.

In the initial population of the proposed DGA, all the individuals are generated randomly according to the above manner. More specifically, the doctor is randomly chosen from the corresponding candidature list for each patient listed in each individual.

**Fig. 3:**
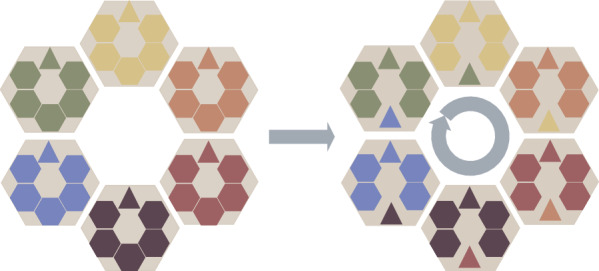
Illustration of distributed framework in DGA

### Distributed framework

As we introduced, GA with the distributed framework has shown its advantages in terms of population diversity, convergence speed, and optimization speed. The initial population is divided into several sub-populations in the distributed framework, each completing the evolutionary process independently. Based on the predefined topology, sub-populations share their elite individuals (e.g., the best individuals) with the predefined migration interval (*MI*). Once one sub-population receives the elite individual from the neighbors in the topology, the current sub-population will randomly select an existing individual (not the best one) to replace.

The proposed DGA uses a distributed framework with a ring communication topology. An example of the distributed framework is given in Fig. 3. As shown in this figure, each big hexagon represents a sub-population. The small triangle and five hexagons represent the best individual and the other five individuals in each big hexagon. During the migration operator, the best individuals in sub-populations are sent to the corresponding neighbor sub-populations according to the ring topology with the predefined migration topology. Afterward, one hexagon in the sub-population is replaced by the triangle, representing one random individual replaced by the best individual.

In DGA, the distributed framework is effective in maintaining population diversity. Thus, the exploration search ability of DGA is guaranteed. Besides, by migrating elite individuals among the sub-populations, the population quality of each sub-population is improved, which helps improve the exploitation search ability of DGA. With the help of the distributed framework and migration operator, DGA is likely to achieve the trade-off between exploration and exploitation during the evolution. Furthermore, the distributed framework helps improve the execution speed of DGA.

### Crossover operator

In genetics, the crossover operator is an algorithmic procedure that encapsulates the phenomena of chromosomal crossover exchange and biological hybridization. For example, the act of recombining and assigning genes on the chromosomes of two parents to form the next generation of humans may combine the dominant genomes of the two parents to produce new individuals more adaptable and closer to the ideal solution via crossing over.

Similarly, the core of GAs is the internal operation of genetic manipulation. By crossover, we mean the function of replacement and recombination of parts of the structure of biparental individuals, resulting in new individuals. The searchability of GAs is greatly improved by crossover. First, general GAs have a mating probability (crossover probability). This mating probability reflects the probability of two selected individuals mating. Each pair of parent individuals produces one or multiple new individuals as the offspring, while the unmated individuals remain unchanged. In the produced child individual, part of the information comes from the father individual, while the left comes from the mother individual.

**Fig. 4:**
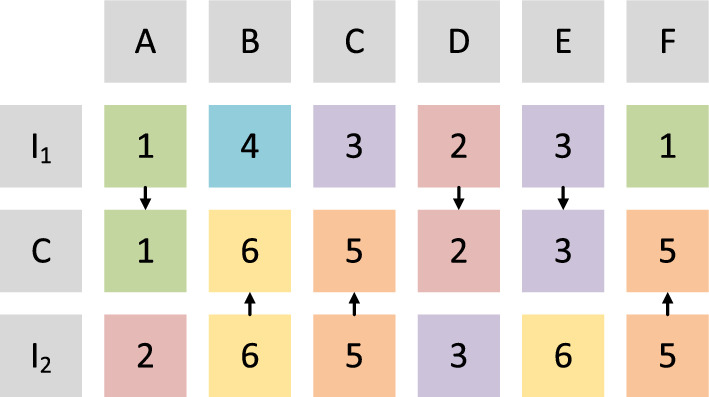
Illustration of crossover operator in DGA

An example of the crossover operator in GA is given in Fig. 4. In the example, two individuals (represented by $\mathrm {I_1}$ and $\mathrm {I_2}$). The information included these two individuals is then exchanged. In this example, each individual includes six genes representing six patients. The values on six genes indicate the assignment of these six patients. For each gene, with the same possibility, one value is randomly chosen from two individuals during the crossover operator. For instance, on the first gene (gene A), the value in individual $\mathrm {I_1}$ is chosen. Thus, in the child individual (represented by C), the value on the first bit is 1. Similarly, on the second gene (gene B), the value in C comes from individual $\mathrm {I_2}$.

**Fig. 5:**
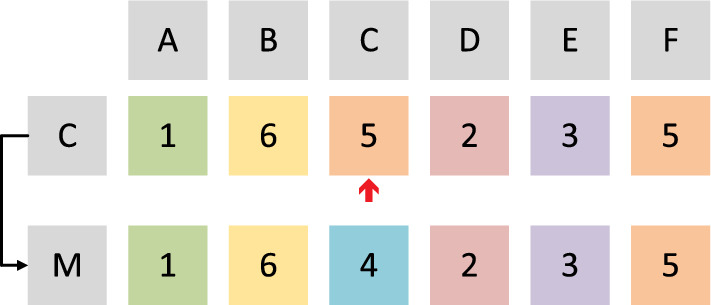
Illustration of mutation operator in DGA

### Mutation operator

There are always individual differences between the parents and offspring of an organism, i.e., differences in the genetic material of different individuals in the same gene pool are called mutations.

The mutation operator’s primary goal is to change the gene values at a specific location in individual strings in the population. The probability of the mutation operator is represented by a constant in the general GA for fixed mutations (the probability of mutation). Based on this probability, a random mutation on the chromosome of a new individual is usually a change of one byte of the chromosome. There are two reasons for introducing mutations into GAs: First, give the GA a local random search function. The variation operator’s local random search capability can speed up the convergence in the optimal solution when the GA approximates the optimal solution neighborhood by the crossover operator. In this case, the variance probability should take a small value. Otherwise, the variation will destroy the building blocks close to the optimal solution. The second is to enable the GA to maintain population diversity and prevent premature convergence. In this case, the convergence probability should take a more meaningful value.

In Fig. 5, an example of the mutation operator is given. With the mutation rate *MR*, each gene of the child individual (represented by C) is randomly adjusted. The third gene (gene C) is chosen randomly in this example. Therefore, its value is randomly adjusted, and its value is changed from 5 to 4.
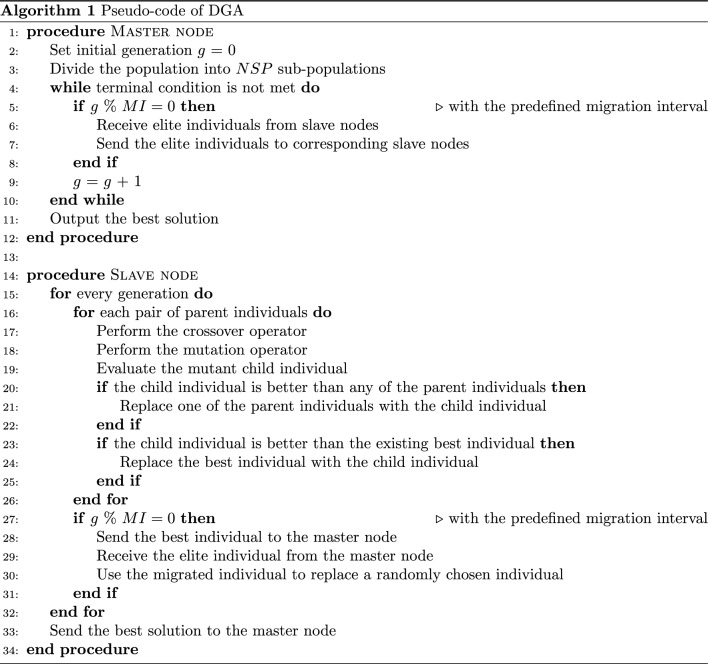


### Overall procedure

The pseudo-code of DGA is given in Algorithm 1. As shown in the pseudo-code, a master–slave model is utilized to implement the DGA algorithm. At the master node, the generation index *g* is set as zero. Then the entire population is divided into *NSP* sub-populations and sent to the corresponding *NSP* slave nodes. With the predefined migration interval *MI*, the master node receives the elite individuals from all the slave nodes. Then it sends these elite individuals to the corresponding slave nodes according to the ring topology. The migration process is executed until the terminal condition is satisfied. Finally, the best solution to the PA problem is outputted.

At the slave node, each sub-population evolves independently. During the evolution, in each generation, for each pair of parent individuals, the crossover operator is executed to exchange the allocation information in parent individuals and generate the child individual. Afterward, the mutation operator is carried out on the child individual to improve the population diversity. After the mutation operator, the mutant child individual is evaluated and compared with the parent individuals by the selection operator. If the mutant child individual is better than any parent individual, one of the parent individuals will be replaced. Otherwise, the mutant child individual will not be kept in the population. Then, the migration operator is carried out with the predefined mutation interval *MI*. Each slave node sends the best individual to the master node and receives one elite individual from the master node. Afterward, one randomly chosen individual in the sub-population that is not the best individual will be replaced by the received migrated individual. Finally, the best individual is returned to the master node.

**Table 1:** Properties of 16 test instances

Test instances	*nP*	*nD*	*T*
$T_1$	100	10	[5, 20]
$T_2$	100	20	[5, 20]
$T_3$	100	30	[5, 20]
$T_4$	100	40	[5, 20]
$T_5$	200	10	[5, 20]
$T_6$	200	20	[5, 20]
$T_7$	200	30	[5, 20]
$T_8$	200	40	[5, 20]
$T_9$	300	10	[5, 20]
$T_{10}$	300	20	[5, 20]
$T_{11}$	300	30	[5, 20]
$T_{12}$	300	40	[5, 20]
$T_{13}$	400	10	[5, 20]
$T_{14}$	400	20	[5, 20]
$T_{15}$	400	30	[5, 20]
$T_{16}$	400	40	[5, 20]

## Experimental setup

This section illustrates the test instances, parameter settings, and algorithm implementation in the following experiments.

In the subsequent experimental studies, 16 test instances are utilized to investigate the performance of the proposed DGA. Table 1 outlines the properties of these test instances, including the number of patients *nP*, the number of doctors *nD*, and the range of estimated diagnosis time *T*.

In the proposed DGA, the sub-population size *SPS* is set as 20; the number of sub-population *NSP* is set as 4; the mutation rate *MR* is set as 0.1; the migration interval *MI* is set as 5. For all the algorithms, the maximum fitness evaluation number is set as $nP \times nD$.

The distributed framework of DGA is implemented by the Message Passing Interface (MPI). Each sub-population is assigned to an independent computation core in the CPU. The communication between sub-populations is implemented by the message passing between CPU cores. DGA and all the compared algorithms in this paper are implemented in C++.

## Experimental result

**Table 2:** TF values of DGA and compared algorithms on all text instances

Test instances	Random		Greedy	DE		GA		DGA	
	Avg	Std	Result	Avg	Std	Avg	Std	Avg	Std
$T_1$	1.28E+01	1.81E+00	2.40E+01	1.11E+01	1.90E+00	6.90E+00	9.27E−01	**5.68E+00** $^\dagger$	8.68E−01
$T_2$	1.49E+01	1.01E+00	1.22E+01	1.24E+01	8.55E−01	7.45E+00	6.43E−01	**6.28E+00** $^\dagger$	6.86E−01
$T_3$	1.40E+01	8.01E−01	9.66E+00	1.13E+01	8.18E−01	7.30E+00	4.94E−01	**5.72E+00** $^\dagger$	6.85E−01
$T_4$	1.37E+01	5.67E−01	8.98E+00	1.14E+01	6.25E−01	8.05E+00	2.76E−01	**6.41E+00** $^\dagger$	3.58E−01
$T_5$	1.91E+01	1.72E+00	4.83E+01	1.60E+01	1.80E+00	9.42E+00	1.26E+00	**7.85E+00** $^\dagger$	9.04E−01
$T_6$	2.03E+01	1.47E+00	2.54E+01	1.64E+01	1.16E+00	1.00E+01	1.05E+00	**7.79E+00** $^\dagger$	8.33E−01
$T_7$	1.91E+01	1.01E+00	1.73E+01	1.50E+01	7.02E−01	9.50E+00	6.93E−01	**7.58E+00** $^\dagger$	5.52E−01
$T_8$	1.86E+01	7.84E−01	1.22E+01	1.51E+01	6.48E−01	9.69E+00	4.59E−01	**7.88E+00** $^\dagger$	6.03E−01
$T_9$	2.08E+01	3.02E+00	8.06E+01	1.63E+01	2.31E+00	1.05E+01	1.23E+00	**8.16E+00** $^\dagger$	1.27E+00
$T_{10}$	2.61E+01	1.91E+00	3.58E+01	1.96E+01	1.50E+00	1.22E+01	8.68E−01	**9.21E+00** $^\dagger$	8.82E−01
$T_{11}$	2.43E+01	1.04E+00	2.54E+01	1.94E+01	1.30E+00	1.22E+01	8.33E−01	**9.97E+00** $^\dagger$	8.09E−01
$T_{12}$	2.07E+01	9.75E−01	1.33E+01	1.72E+01	8.47E−01	1.11E+01	5.24E−01	**9.19E+00** $^\dagger$	4.46E−01
$T_{13}$	2.24E+01	3.51E+00	9.62E+01	1.91E+01	3.23E+00	1.12E+01	1.64E+00	**8.51E+00** $\dagger$	1.18E+00
$T_{14}$	2.93E+01	2.25E+00	3.89E+01	2.24E+01	2.05E+00	1.37E+01	9.76E−01	**1.06E+01** $^\dagger$	9.58E−01
$T_{15}$	2.78E+01	1.28E+00	2.61E+01	2.15E+01	1.44E+00	1.34E+01	9.04E−01	**1.09E+01** $^\dagger$	6.53E−01
$T_{16}$	2.57E+01	1.18E+00	1.91E+01	2.03E+01	1.03E+00	1.32E+01	9.04E−01	**1.06E+01** $^\dagger$	6.58E−01

^†^indicates that the difference among the compared results is significant based on the Wilcoxon rank-sum test with a 5% level

**Fig. 6:**
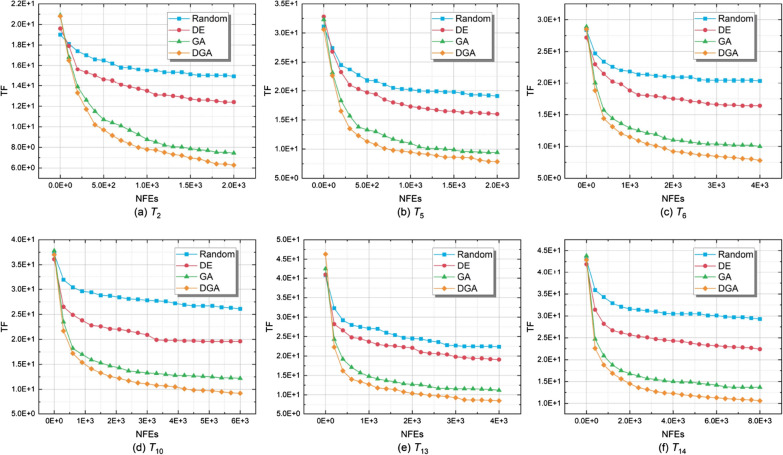
Convergence curves of DGA and compared algorithms on six typical test instances

### Comparison with existing approaches

To verify the performance of the proposed DGA, it is compared with three existing algorithms, i.e., Random, Greedy, differential evolution (DE) [54], and GA [43]. These algorithms are described as follows:

1. Random: This algorithm uses a random manner to solve the PA problem. Random solutions are continuously generated and compared with the best solution. The best solution is replaced once a more competitive solution is generated.
2. Greedy: This algorithm uses a greedy manner to solve the PA problem. Each patient is greedily allocated to a doctor.
3. DE [54]: This DE algorithm utilizes the “DE/best/1” mutation schema to generate the mutant individuals, which can help accelerate the exploitation search ability during the optimization of the PA problem.
4. GA [43]: In this algorithm, a GA is specifically designed for the PA problem, including the representation manner, crossover operator, and mutation operator.

Table 2 lists the mean (Avg) and standard deviation (Std) of TF values (defined in Sect. 2) over 25 independent runs. Therefore, a lower TF value indicates that the corresponding algorithm can provide better optimization performance with regard to the balance of doctors’ diagnosis time. The best results (i.e., the lowest Avg values) in Table 2 are highlighted in boldface. The Greedy approach only lists its results since it can generate deterministic results. The proposed DGA can achieve significant advantages on all 16 test instances. The benefits of DGA in heuristic strategies are confirmed compared to Random. With the help of the crossover and mutation operators, information allocation among different individuals is effectively exchanged, and more competitive individuals are generated and inserted into the population. Compared with Greedy, the stronger feasibility of DGA in population diversity is verified. The greedy technique is more likely to get trapped by the local optima during the PA problem optimization. Unlike the Greedy approach, the population diversity of DGA can effectively guarantee the exploration search ability of DGA. The benefit of DGA in discrete-domain optimization is demonstrated when compared to DE. The mutation strategy of DE, such as “DE/best/1”, is efficient in the continuous-domain calculation. For this discrete PA problem, its mutation strategies are challenging to transfer information among individuals. Compared with GA, the advantage of the distributed framework in DGA is verified. Moreover, with the architecture of the distribution framework, the population diversity in DGA is maintained. Furthermore, the migration of elite individuals among sub-populations accelerates the optimization process. As a result, the advantage of convergence speed is based on the ring topology.

Besides, the Wilcoxon rank-sum test with a 0.05 level is utilized to investigate the performance of these algorithms in a statistical sense. In Table 2, the symbol $^\dagger$ shows that the corresponding result is significantly better than the other compared results. The advantage of DGA obtained in all the test instances is significant.

According to the problem formulation in Sect. 2, the time complexity of calculating a given solution to the PA problem is $\mathcal {O}(nP)$. Therefore, the time complexity of the proposed DGA is $\mathcal {O}(nP^2\times nD)$. Similarly, the time complexity of Random, DE, and GA is $\mathcal {O}(nP^2\times nD)$, the same as DGA. Different from these algorithms, the time complexity of the Greedy method is $\mathcal {O}(nP\times nD)$. Although the Greedy method is of lower time complexity, due to the limitation of search diversity, its optimization performance is significantly worse than the proposed DGA. Regarding the space complexity, the space complexity of Random and Greedy is $\mathcal {O}(nP\times nD)$, while the space complexity of DE, GA, and the proposed DGA is $\mathcal {O}(nP\times (nD+SPS))$.

In Fig. 6, the convergence curves of Random, DE, GA, and DGA on six typical test instances are plotted. A line with unique color indicates each approach. The number of fitness evaluations is indicated on the horizontal axis, and the value of TF is represented on the vertical axis for each point on the line. The Greedy approach is not given in the figure since no eligible solution is generated during the greedy construction. Compared with the Random approach, the advantage of DGA in search efficiency is verified. Furthermore, with the help of the population crossover and mutation operators, DGA is more likely to achieve the trade-off between exploration and exploitation. Compared with DE, the advantage of DGA in discrete-domain optimization is verified. In addition, compared with GA, the advantage of DGA in information exchange efficiency and population diversity is shown. In summary, DGA achieves the best convergence performance in all six test instances.

**Table 3:** TF values of DGA and three variants on all text instances

Test instances	DGA-no-crossover		DGA-no-mutation		DGA-no-distributed		DGA	
	Avg	Std	Avg	Std	Avg	Std	Avg	Std
$T_1$	6.50E+00	9.99E−01	6.91E+00	1.07E+00	6.90E+00	9.27E−01	**5.68E+00**$^\dagger$	8.68E−01
$T_2$	7.57E+00	5.36E−01	7.67E+00	8.24E−01	7.45E+00	6.43E−01	**6.28E+00**$^\dagger$	6.86E−01
$T_3$	7.18E+00	5.86E−01	7.04E+00	7.05E−01	7.30E+00	4.94E−01	**5.72E+00**$^\dagger$	6.85E−01
$T_4$	7.87E+00	4.23E−01	7.79E+00	4.42E−01	8.05E+00	2.76E−01	**6.41E+00**$^\dagger$	3.58E−01
$T_5$	8.39E+00	1.15E+00	1.02E+01	1.64E+00	9.42E+00	1.26E+00	**7.85E+00**$^\dagger$	9.04E−01
$T_6$	9.23E+00	1.09E+00	9.60E+00	9.93E−01	1.00E+01	1.05E+00	**7.79E+00**$^\dagger$	8.33E−01
$T_7$	8.72E+00	6.58E−01	8.71E+00	8.33E−01	9.50E+00	6.93E−01	**7.58E+00**$^\dagger$	5.52E−01
$T_8$	9.05E+00	6.25E−01	9.25E+00	4.95E−01	9.69E+00	4.59E−01	**7.88E+00**$^\dagger$	6.03E−01
$T_9$	9.44E+00	1.37E+00	1.05E+01	1.30E+00	1.05E+01	1.23E+00	**8.16E+00**$^\dagger$	1.27E+00
$T_{10}$	1.06E+01	1.17E+00	1.16E+01	8.93E−01	1.22E+01	8.68E−01	**9.21E+00**$^\dagger$	8.82E−01
$T_{11}$	1.10E+01	8.59E−01	1.15E+01	9.84E−01	1.22E+01	8.33E−01	**9.97E+00**$^\dagger$	8.09E−01
$T_{12}$	1.03E+01	5.75E−01	1.04E+01	6.27E−01	1.11E+01	5.24E−01	**9.19E+00**$^\dagger$	4.46E−01
$T_{13}$	1.05E+01	1.39E+00	1.15E+01	1.79E+00	1.12E+01	1.64E+00	**8.51E+00**$^\dagger$	1.18E+00
$T_{14}$	1.24E+01	1.05E+00	1.30E+01	9.91E−01	1.37E+01	9.76E−01	**1.06E+01**$^\dagger$	9.58E−01
$T_{15}$	1.24E+01	8.24E−01	1.26E+01	7.41E−01	1.34E+01	9.04E−01	**1.09E+01**$^\dagger$	6.53E−01
$T_{16}$	1.23E+01	7.32E−01	1.22E+01	5.55E−01	1.32E+01	9.04E−01	**1.06E+01**$^\dagger$	6.58E−01

^†^indicates that the difference among the compared results is significant based on the Wilcoxon rank-sum test with a 5% level

### Impact of proposed components

In this section, we will experiment to verify the impact of the proposed components in the DGA. Besides the proposed DGA, we have implemented three variants.

1. DGA-no-crossover: This variant is implemented by removing the crossover operator from DGA.
2. DGA-no-mutation: In this variant, the mutation operator is removed. Accordingly, the crossover operator and distributed framework are kept.
3. DGA-no-distributed: In this variant, the proposed distributed framework is proposed. Therefore, this variant is implemented serially.

Table 3 lists the average (Avg) and standard deviation (Std) values of TF (defined in Sect. 2) obtained by three variants and DGA. The best results (i.e., the lowest Avg values) in all the test instances are labeled in boldface. Overall, the complete-version DGA can outperform the compared variants on all 16 test instances. Compared with DGA-no-crossover, DGA shows its advantage in terms of the crossover operator, effectively exchanging allocation between parent individuals. Compared with DGA-no-mutation, DGA shows its advantage in terms of the mutation operator, which can effectively improve population diversity. Finally, compared with the variant DGA-no-distributed, DGA shows the advantage of the distributed framework, which can effectively balance the exploration and exploitation searching abilities.

Besides, the Wilcoxon rank-sum (significance level 0.05) is employed to verify DGA’s advantage in a statistical sense. As shown in the table, the symbol $^\dagger$ shows that the labeled results are significantly better than the compared results. In all 16 test instances, the advantages of the complete DGA are significant.

## Conclusion

In this paper, a DGA has been proposed to optimize the balance of PA schedules. Each individual in the proposed DGA represents a solution for the PA optimization problem. Furthermore, three operators in the proposed DGA, i.e., crossover, mutation, and selection, have been utilized to improve the competitiveness of these solutions. The distributed framework in the proposed DGA helps improve population diversity and scalability. Through the analysis of the experimental results, we have verified that the proposed DGA effectively optimizes the PA problem. In addition, we have verified the effectiveness of all the proposed components.

In the future, it would be crucial to include more objectives in the PA problem. Thus, some practical multi-objective optimization algorithms should be designed accordingly.

## References

[1] Du J, Michalska S, Subramani S (2019). Neural attention with character embeddings for hay fever detection from twitter. Health Inf Sci Syst.

[2] He J, Rong J, Sun L (2020). A framework for cardiac arrhythmia detection from IoT-based ECGs. World Wide Web.

[3] Hong W, Yin J, You M, et al. Graph intelligence enhanced bi-channel insider threat detection. In: Network and System Security. Springer Nature Switzerland; 2022. pp. 86–102. 10.1007/978-3-031-23020-2_5.

[4] Jiang H, Zhou R, Zhang L (2018). Sentence level topic models for associated topics extraction. World Wide Web.

[5] Siuly S, Alcin OF, Kabir E (2020). A new framework for automatic detection of patients with mild cognitive impairment using resting-state EEG signals. IEEE Trans Neural Syst Rehabil Eng.

[6] Vimalachandran P, Liu H, Lin Y (2020). Improving accessibility of the Australian my health records while preserving privacy and security of the system. Health Inf Sci Syst.

[7] Yin J, Tang M, Cao J (2023). Knowledge-driven cybersecurity intelligence: software vulnerability coexploitation behavior discovery. IEEE Trans Ind Inf.

[8] Lee J, Park J, Wang K, et al. The use of telehealth during the coronavirus (COVID-19) pandemic in oral and maxillofacial surgery: a qualitative analysis. In: ICST Transactions on Scalable Information Systems; 2021. p. 172361. 10.4108/eai.2-12-2021.172361.

[9] Wang H, Li Y, Li Y (2018). Patient assignment scheduling in a cloud healthcare system based on petri net and greedy-based heuristic. Enterp Inf Syst.

[10] Sarki R, Ahmed K, Wang H, et al. Convolutional neural network for multi-class classification of diabetic eye disease. In: ICST Transactions on Scalable Information Systems; 2021. p. 172436. 10.4108/eai.16-12-2021.172436.

[11] Singh R, Zhang Y, Wang H, et al. Investigation of social behaviour patterns using location-based data: a melbourne case study. In: ICST Transactions on Scalable Information Systems; 2020. p. 166767. 10.4108/eai.26-10-2020.166767.

[12] Yin J, Tang M, Cao J (2020). Apply transfer learning to cybersecurity: predicting exploitability of vulnerabilities by description. Knowl-Based Syst.

[13] You M, Yin J, Wang H (2022). A knowledge graph empowered online learning framework for access control decision-making. World Wide Web.

[14] Pandey D, Wang H, Yin X (2022). Automatic breast lesion segmentation in phase preserved DCE-MRIs. Health Inf Sci Syst.

[15] Sarki R, Ahmed K, Wang H (2020). Automated detection of mild and multi-class diabetic eye diseases using deep learning. Health Inf Sci Syst.

[16] Supriya S, Siuly S, Wang H (2020). Automated epilepsy detection techniques from electroencephalogram signals: a review study. Health Inf Sci Syst.

[17] Chenthara S, Ahmed K, Wang H (2020). Healthchain: a novel framework on privacy preservation of electronic health records using blockchain technology. PLoS ONE.

[18] Hu H, Li J, Wang H, et al. Combined gene selection methods for microarray data analysis. In: Lecture Notes in Computer Science. Springer Berlin Heidelberg; 2006. pp. 976–83. 10.1007/11892960_117.

[19] Malhotra V, Sandhu M. Improved ECG based stress prediction using optimization and machine learning techniques. In: ICST Transactions on Scalable Information Systems; 2018. p. 169175. 10.4108/eai.6-4-2021.169175.

[20] Nigam K, Godani K, Sharma D (2021). An improved approach for stress detection using physiological signals. ICST Trans Scalable Inf Syst.

[21] Sarki R, Ahmed K, Wang H (2020). Automatic detection of diabetic eye disease through deep learning using fundus images: a survey. IEEE Access.

[22] Hu H, Li J, Wang H, et al. A maximally diversified multiple decision tree algorithm for microarray data classification. In: Proceedings of the 2006 Workshop on Intelligent Systems for Bioinformatics. Australian Computer Society; 2006. pp. 35–38.

[23] Khalil F, Li J, Wang H (2009). An integrated model for next page access prediction. Int J Knowl Web Intell.

[24] Peng M, Zhu J, Wang H (2018). Mining event-oriented topics in microblog stream with unsupervised multi-view hierarchical embedding. ACM Trans Knowl Discov Data.

[25] Subramani S, Wang H, Vu HQ (2018). Domestic violence crisis identification from facebook posts based on deep learning. IEEE Access.

[26] Hossain NUI, Debusk H, Hasan MM. Reducing patient waiting time in an outpatient clinic: a discrete event simulation (des) based approach. In: Proceedings of the IIE Annual Conference. Institute of Industrial and Systems Engineers (IISE); 2017. pp. 241–46.

[27] Munavalli JR, Rao SV, Srinivasan A (2019). Integral patient scheduling in outpatient clinics under demand uncertainty to minimize patient waiting times. Health Inf J.

[28] Ge YF, Cao J, Wang H, et al. A benefit-driven genetic algorithm for balancing privacy and utility in database fragmentation. In: Proceedings of the Genetic and Evolutionary Computation Conference. ACM; 2019. pp 771–76. 10.1145/3321707.3321778.

[29] Srinivas M, Patnaik L (1994). Genetic algorithms: a survey. Computer.

[30] Chen ZG, Zhan ZH, Wang H (2020). Distributed individuals for multiple peaks: a novel differential evolution for multimodal optimization problems. IEEE Trans Evol Comput.

[31] Ge YF, Yu WJ, Zhang J. Diversity-based multi-population differential evolution for large-scale optimization. In: Proceedings of the 2016 on Genetic and Evolutionary Computation Conference Companion. ACM; 2016. 10.1145/2908961.2908995.

[32] Ge YF, Orlowska M, Cao J (2022). MDDE: multitasking distributed differential evolution for privacy-preserving database fragmentation. VLDB J.

[33] Wang ZJ, Zhan ZH, Lin Y (2020). Automatic niching differential evolution with contour prediction approach for multimodal optimization problems. IEEE Trans Evol Comput.

[34] Ge YF, Yu WJ, Zhan ZH, (2018) Competition-based distributed differential evolution. In: IEEE Congress on Evolutionary Computation (CEC). IEEE; 2018. 10.1109/cec.2018.8477758.

[35] Ge YF, Yu WJ, Cao J (2021). Distributed memetic algorithm for outsourced database fragmentation. IEEE Trans Cybern.

[36] Mirjalili S (2018). Evolutionary algorithms and neural networks.

[37] Ge YF, Wang H, Cao J, et al. An information-driven genetic algorithm for privacy-preserving data publishing. In: Web Information Systems Engineering—WISE 2022. Springer International Publishing; 2022. pp. 340–354. 10.1007/978-3-031-20891-1_24.

[38] Ge YF, Zhan ZH, Cao J (2022). DSGA: a distributed segment-based genetic algorithm for multi-objective outsourced database partitioning. Inf Sci.

[39] Huang T, Gong YJ, Kwong S, et al. A niching memetic algorithm for multi-solution traveling salesman problem. In: IEEE Transactions on Evolutionary Computation; 2019. pp. 1–1. 10.1109/tevc.2019.2936440.

[40] Ge YF, Cao J, Wang H, et al. Distributed differential evolution for anonymity-driven vertical fragmentation in outsourced data storage. In: Web Information Systems Engineering—WISE 2020. Springer International Publishing; 2020. pp. 213–26. 10.1007/978-3-030-62008-0_15.

[41] Ge YF, Orlowska M, Cao J (2021). Knowledge transfer-based distributed differential evolution for dynamic database fragmentation. Knowl-Based Syst.

[42] Li JY, Du KJ, Zhan ZH (2022). Distributed differential evolution with adaptive resource allocation. IEEE Trans Cybern.

[43] Pang X, Ge YF, Wang K. Genetic algorithm for patient assignment optimization in cloud healthcare system. In: Health Information Science. Springer Nature Switzerland; 2022, pp. 197–208. 10.1007/978-3-031-20627-6_19.

[44] Barros PP, Olivella P (2005). Waiting lists and patient selection. J Econ Manag Strategy.

[45] Takakuwa S, Wijewickrama A. Optimizing staffing schedule in light of patient satisfaction for the whole outpatient hospital ward. In: Winter Simulation Conference. IEEE; 2008. 10.1109/wsc.2008.4736230.

[46] Patrick J, Puterman ML, Queyranne M (2008). Dynamic multipriority patient scheduling for a diagnostic resource. Oper Res.

[47] Gijo EV, Antony J (2013). Reducing patient waiting time in outpatient department using lean six sigma methodology. Qual Reliab Eng Int.

[48] Mardiah FP, Basri MH (2013). The analysis of appointment system to reduce outpatient waiting time at Indonesia’s public hospital. Hum Resour Manag Res.

[49] Chawasemerwa T, Taifa I, Hartmann D. Development of a doctor scheduling system: a constraint satisfaction and penalty minimisation scheduling model. Int J Res Ind Eng 2018;7(4):396–422. 10.22105/riej.2018.160257.1068

[50] Conforti D, Guerriero F, Guido R (2007). Optimization models for radiotherapy patient scheduling. 4OR.

[51] Yadav AS, Ahlawat N, Sharma N, et al. Healthcare system of inventory control for blood bank storage with reliability applications using genetic algorithm. Adv Math. 2020;9(7):5133–42. 10.37418/amsj.9.7.80.

[52] Ahmed R, Zayed T, Nasiri F (2020). A hybrid genetic algorithm-based fuzzy Markovian model for the deterioration modeling of healthcare facilities. Algorithms.

[53] Mutingi M, Mbohwa C. Home healthcare worker scheduling: a group genetic algorithm approach. In: Proceedings of the World Congress on Engineering 2013; 2013.

[54] Price KV, Zelinka I, Snasael V, Abraham A (2013). Differential evolution. Handbook of optimization.

